# Net-Immobilization of *β*-glucosidase on Nonwoven Fabrics to Lower the Cost of “Cellulosic Ethanol” and Increase Cellulose Conversions

**DOI:** 10.1038/srep23437

**Published:** 2016-03-24

**Authors:** Xing Zhu, Bin He, Changwen Zhao, Rong Fan, Lihua Zhang, Guan Wang, Yuhong Ma, Wantai Yang

**Affiliations:** 1 State Key Laboratory of Chemical Resource Engineering, Beijing University of Chemical Technology, Beijing 100029, China; 2 Beijing Laboratory of Biomedical Materials, Beijing University of Chemical Technology, Beijing 100029, China; 3 Key Laboratory of Carbon Fiber and Functional Polymers, Ministry of Education, Beijing University of Chemical Technology, Beijing 100029, China

## Abstract

The main limitation preventing the use of enzymatic cellulosic ethanol in industrial production is its higher cost which is mainly due to the elevated price of *β*-glucosidase (BG). Herein, we report on a simple strategy for the *in-situ* encapsulation of BG for repeated cellulosic ethanol production. In this strategy, BG was net-immobilized into a poly(ethylene glycol) (PEG) net-cloth layer on a PP nonwoven fabric by way of the visible light-induced surface controlled/living graft cross-linking polymerization. The visible light and mild reaction conditions could ensure the activity retention of BG during immobilization, while the non-swelling uniform net-mesh formed by living cross-linking polymerization could prevent the leakage of BG effectively (at the immobilization rate of more than 98.6% and the leakage rate of only 0.4%). When the BG-loaded fabric was used in combination with free cellulase (CEL), the results of the catalytic reaction demonstrated that these BG-loaded fabrics could not only give a 40% increase in cellulose conversions but also be reused for more than fifteen batches without losing the activity. These BG-loaded fabrics with characteristics including easy separation, excellent operation stability, a low cost of the polymeric matrix and a simple fabrication process are particularly interesting for a future bio-fuel production strategy.

The growing demand for energy combined with diminishing fossil fuel reserves have stimulated a tremendous interest in finding alternative renewable energy sources^1^,^2^.“Cellulosic ethanol”, obtained by turning all kinds of plant material (from waste straw to useless wood) into fuels^3^,^4^,^5^,^6^,^7^,^8^,^9^,^10^,^11^, is a promising sustainable substitution for fossil fuel.

One mild and green way to produce “cellulosic ethanol” is to convert cellulose into fermentable sugars by a complex enzyme hydrolysis and then ferment the sugars into ethanol^12^,^13^,^14^,^15^. But the main limitation preventing this enzymatic method to be applied in industrial production is the higher costs as opposed to corn ethanol and sugarcane ethanol, mainly due to the elevated price of enzymes^16^,^17^,^18^,^19^,^20^,^21^.

Cellulase (CEL) is a complex enzyme system that includes endo-glucanase (EG, EC 3.2.1.4), cellobiohydrolase (CBH, EC 3.2.1.91), and *β*-glucosidase (BG, EC 3.2.1.21). The hydrolysis of cellulose comes from the synergistic effect of the multi-enzyme systems. Firstly, EG hydrolyzes accessible regions on cellulose chains to provide new sites for CBH catalyzing. And then the intermediate products were cut to cellobiose units. Finally, BG catalyzes the hydrolysis of cellobiose, which is known to be a strong inhibitor of the activities of the other two enzymes^22^,^23^.

Due to the fact that the content of BG is much lower than that of the other two enzymes of the CEL system, the rate that converting cellobiose into glucose is relatively slow^24^. Thus adding extra BG to the hydrolysis system can significantly improve the conversions^25^,^26^,^27^. In particular, among the CEL system, the cost of BG is 100 times higher than that of the other two enzymes. Therefore, to develop an efficient “BG immobilization” technique and permit multiple reuse without loss of activity would greatly reduce the cost of cellulosic ethanol^28^,^29^,^30^,^31^,^32^,^33^.

Various methods have been reported for BG immobilization, including adsorption, covalent binding and entrapment^34^,^35^,^36^,^37^,^38^. But up to now, the development of a cost-efficient way to reduce the leakage of BG after immobilization and enhance the separation after reaction still remains very challenging but would definitely improve the reuse times and the operational stability. To address the above mentioned issue, many efforts have been made to improve the operational stabilities of the immobilized BG. For example, Zheng *et al*.^39^ used chitosan and Fe_3_O_4_ to prepare magnetic chitosan microspheres for the immobilization of BG, and after magnetic separation, it could be used for eight rounds of hydrolysis. Figueira *et al*.^40^ entrapped BG in either sol–gel or PVA–gel (Lentikats^®^) supports for hydrolyzing cellobiose. Compared with the sol–gel method, the BG immobilized in Lentikats^®^ was proved to be a more scalable method with the advantage of particles being easily separated from the reaction medium. And they demonstrated that Lentikats^®^–entrapped glucosidase could be used in 19 consecutive batch runs for cellobiose hydrolysis, without noticeable decrease in product yield. Tan *et al*.^41^ synthesized the stable polymer hybrid matrix κ-carrageenan beads to immobilize BG. The immobilized BG could still retain about 75% of its initial activity after repeated 12 batch runs. Wei *et al*.^42^ prepared mercaptopropyl-functionalized M-TiO_2_ (SH-M-TiO_2_) as the carrier for the BG immobilization, the conversion rates of cellobiose were consistently around 90% in 10 batch runs.

Herein, different from the above methods, we report on a novel and very simple “*in-situ* BG net-immobilization” method by a one-step “visible light-induced surface graft cross-linking polymerization” with poly(ethylene glycol) diacrylate (PEGDA) as the monomer and a polypropylene (PP) nonwoven fabric as the supporting matrix^43^. The final product formed a two-layer composite sheet structure where BG was trapped in the network of PEG’s molecular net-cloth and grafted onto the surface of the PP matrix. This design has the following advantages: 1) most of the hydrogels are sensitive to the environment, which can cause uncontrolled variations on their performance. But the PEG molecular net-cloth we used has the non-swelling feature in aqueous phase which can effectively prevent the enzyme leakage; 2) the mild polymerization conditions (i.e. visible light and room temperature) are ideal for *in situ* encapsulation of delicate biomolecules such as enzymes; 3) most of the hydrogel networks suffering from a lack of sufficient mechanical strength thus are easy to be damaged during practical use, while grafting the net-cloth onto supporting substrate (here is the non-woven fabric) can eliminate this drawback and ensure the operational stability^44^; 4) compared with other carriers such as particles, the polymeric sheets can be more easily separated for reusing; 5) this strategy is applicable to any C-H containing surface and can be easily tailored for a broad range of applications on other substrates^45^.

## Results and Discussions

**Figure 1:**
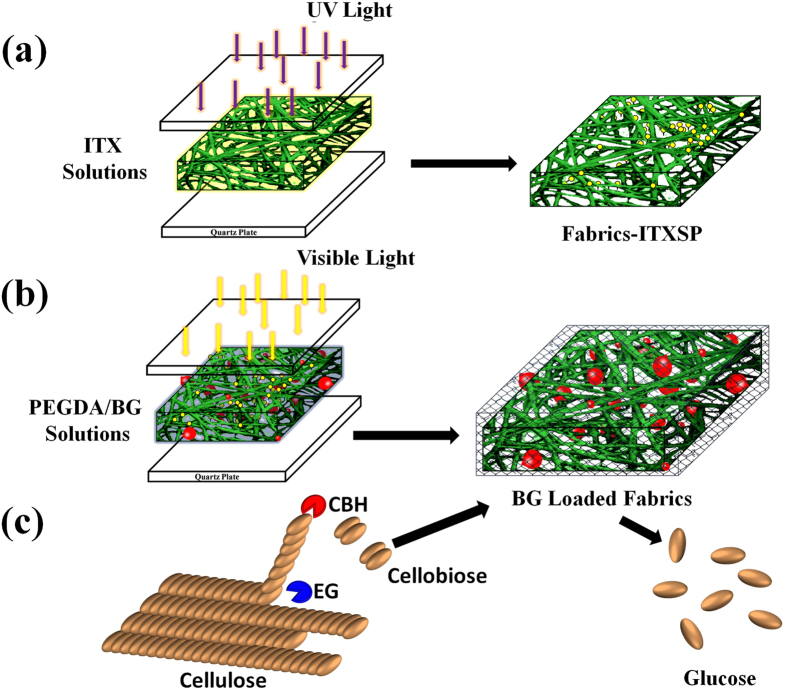
Schematic Illustration of the Immobilization Processes of BG on a PP Nonwoven Fabric and the Hydrolyzed Reaction Catalyzed by a BG-Loaded Fabric. (**a**) ITXSP was coupled with the nonwoven fabric via a UV-induced photo-reduction reaction. (**b**) Visible light-induced surface graft cross-linking polymerization of PEGDA/BG solutions with a sandwich structure to immobilize BG. (**c**) The use of the free cellulase and BG-loaded fabric to convert cellulose to glucose.

The process of *in-situ* immobilization of BG into the PEG net-cloth grafted onto the PP nonwoven fabric is shown in Fig. 1. In Fig. 1a, the photoinitiator thioxanthone (ITX) was first planted on a fabric under UV irradiation by abstracting hydrogen and performing a coupling reaction to form the thioxanthone-semipinacol (ITXSP) dormant groups. These groups can be triggered by visible light giving rise to surface radicals and ITXSP radicals. In Fig. 1b, we see the surface carbon radical initiating the graft polymerization of PEGDA, while the ITXSP radical mediated the polymerization by reversible deactivation of the propagating radicals. When the BGs were added to a monomer solution of PEGDA, they became *in-situ* embedded into the newly formed PEG net-cloth during the graft cross-linking polymerization. Figure 1c shows how cellulose became hydrolyzed by free CEL to produce cellobiose and glucose, and the extra amount of cellobiose was further converted into glucose, mediated by the BG-loaded fabrics.

**Figure 2:**
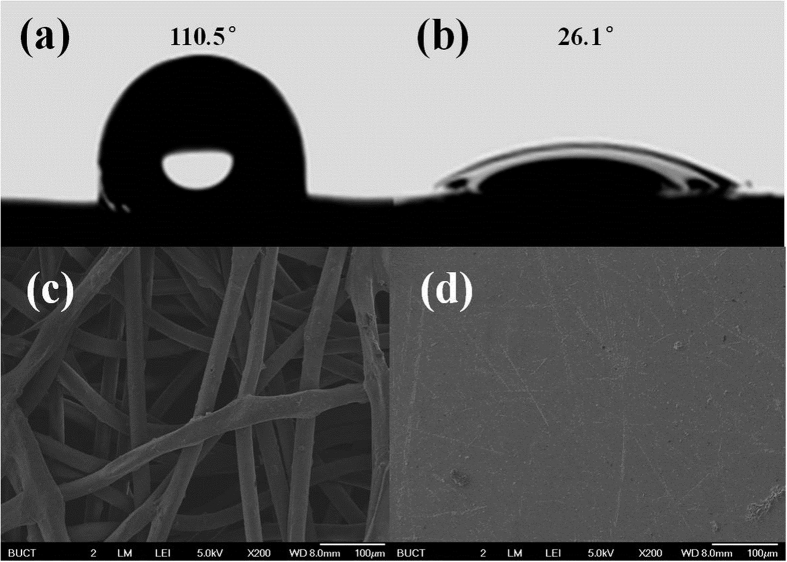
Contact angles of (**a**) nonwoven fabric-ITXSP surface, (**b**) BG-loaded fabric surface. SEM images of (**c**) PP nonwoven fabric and (**d**) BG-loaded fabric. BG-loaded fabrics were made as follows: an 800 μL BG solution was mixed with 600 μL PEGDA via shaking, after which the solutions were cast onto the fabric-ITXSP. The fabric was sandwiched between two quartz plates to spread the PEGDA/BG solution. The time of polymerization was 90 min under the xenon lamp.

The changes in contact angles between the fabric-ITXSP and BG-loaded fabric are shown in Fig. 2a,b. The contact angle decreased significantly (from 110.5° to 26.1°) after grafting of the PEG net-cloth to the fabric, and it seemed obvious that the change in contact angles resulted from the introduction of the highly hydrophilic PEG net-cloth.

The surface morphology of the non-woven fabrics was studied using scanning electron microscopy (SEM). Figure 2c,d present SEM images of the non-woven fabric and BG-loaded fabric. The significant differences between Fig. 2c,d clearly proved that the P(PEGDA)/BG layer could be uniformly grafted onto the nonwoven fabric.

**Figure 3:**
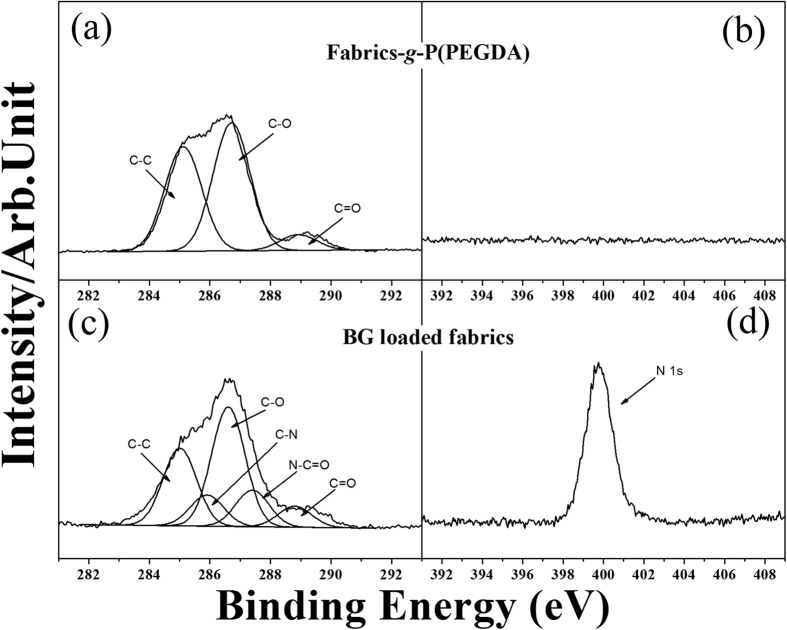
XPS C 1s and N 1s core-level spectra of (**a,b**) Fabric-*g*-P(PEGDA) and (**c,d**) BG-loaded fabric.

To further confirm the successful preparation of the BG-loaded fabric, X-ray photoelectron spectroscopy (XPS) was used to determine the chemical composition of the modified nonwoven fabric. Figure 3 shows the C 1s and N 1 s core-level spectra of fabric-*g*-P(PEGDA) (fabric grafted with P(PEGDA) without BG as a control) and of the BG-loaded fabric. As can be seen in Fig. 3a, the C 1s core-level spectrum of fabric-*g*-P(PEGDA) can be curve-fitted into three peak components with binding energies at 285.0 eV for the C−H and C−C species, 286.6 eV for the C−O species, and 288.8 eV for the O=C species^46^. This result demonstrated that the P(PEGDA) net-cloth had been successfully grafted onto the nonwoven fabric. Moreover, XPS also provided a feasible method to verify fabric-g-P(PEGDA) and BG loaded fabrics. In Fig. 3c, the C 1 s core-level spectra of the surfaces of the BG-loaded fabric had two new peaks at about 285.9 and 287.4 eV, attributed to the C−N and O=C−N species which were associated with the amino and peptide bonds in BG. Additionally, an N 1s signal at a binding energy of about 399.5 eV, characteristic of covalently bonded nitrogen, can be seen in Fig. 3d. However, no N 1 s signal was detected for the fabric-*g*-P(PEGDA) surfaces (Fig. 3b). All the above results indicated that BG had been successfully embedded into the P(PEGDA) net-cloth on the fabric. The successful entrapment of BG was also confirmed by the microscope photo and AFM images, which are shown in Figure S1.

**Figure 4:**
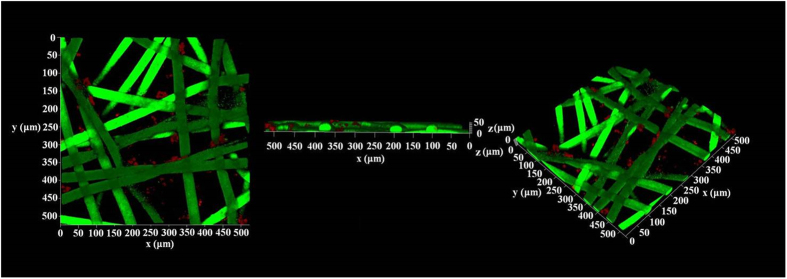
CLSM images of the BG-loaded fabric dyed with FUN^®^ 1 and Calcofluor^®^. A BG-loaded fabric with fluorescent dye was prepared as follows: the BG loaded fabric was immersed into 100 mL of Na-HEPES buffer (10 mM, pH = 7.2), after which 100 μL FUN^®^ 1 and 500 μL Calcofluor^®^ solutions were added to the buffer. After 60 min at room temperature, the BG-loaded fabric was taken out from the solution and thoroughly washed with Na-HEPES buffer to remove the fluorescent agent without interaction.

Based on the molecular weight of PEGDA 575, the average volume of each PEG grid should be approximately 27 nm^3^ 43, while the size of the BG molecules had been reported to be less than 25 nm^3^ 47. Following this routine method for increasing the size of the enzymes, to prevent BG from leaching out of the PEG networks on the fabric, glutaraldehyde (GA) was used to cross-link BG, thus increasing the size of the enzyme so that it could form enzyme clusters. To clear up the distribution of BG clusters in the PEG net-cloth, we used FUN^®^ 1 and Calcofluor^®^ to stain the BG-loaded fabric. Figure 4 represents the 3D fluorescence images of the BG-loaded fabrics characterized by confocal laser scanning microscope (CLSM). It could be clearly observed that the fibers of the non-woven fabric were dyed green and that the BG clusters were dyed red. The PEG net-cloth on the other hand was not dyed by any of the two agents. The fluorescence indicated that most of the BG clusters were distributed near the fibers of the nonwoven fabric.

**Figure 5:**
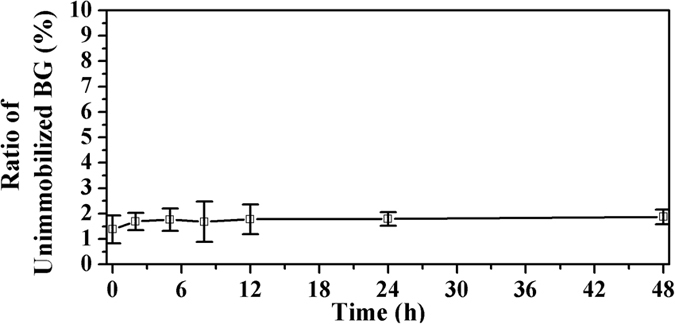
Release behavior of the cross-linked BG (0.25% (v/v) of GA) immobilized in the PEG net-cloth on the nonwoven fabric.

For this system comprising an *in-situ* encapsulating enzyme in a PEG network, two parameters are essential: the ratio of enzymes entrapped in the PEG-network during the reaction process and the ratio of enzymes that had leached from the PEG-network during the working state. To ascertain these two parameters, we followed the data of the BG concentration in citric acid buffer after the BG-loaded fabric had been incubated in this buffer for a few hours. Figure 5 shows the plot of the unimmobilization ratio as a function of time. The data at 0 h represented the unimmobilized BG in the grafting process. When 0.25% (v/v) GA was used to cross-link BG, about 1.4% of unimmobilized BG was observed after the graft reaction, which meant that the ratio of enzymes entrapped in the PEG-network during the reaction process was 98.6%. After immersing the fabric in buffers for 48 h, about 1.8% of the BG was unimmobilized. If we substract the amount of BG unimmobilized after the graft reaction, the ratio of enzymes leached from the PEG-network after immersion in buffers is about 0.4%. Compared with the traditional hydrogels, this uniform and non-swelling net-cloth can retain most of the enzymes without leaking them.

The activity of immobilized BG is another important indicator for evaluating the performance of this catalytic fabric. The BG activity was measured as the amount of *p*-nitrophenol released from *p*-nitrophenyl-*β*-D-glucopyranoside (*p*-NPG) using a calibration curve at 405 nm^43^. The activities of the BG-loaded fabric were found to be 15.8 U/g. It should be noted that some BG may be covalently bonded to the net-cloth *via* Michael addition between lysine or cysteine residues and acrylate groups of PEGDA, which may cause some deactivation of the immobilized BG, but 15.8 U/g is still high enough to ensure the application of glucose production^43^.

**Figure 6:**
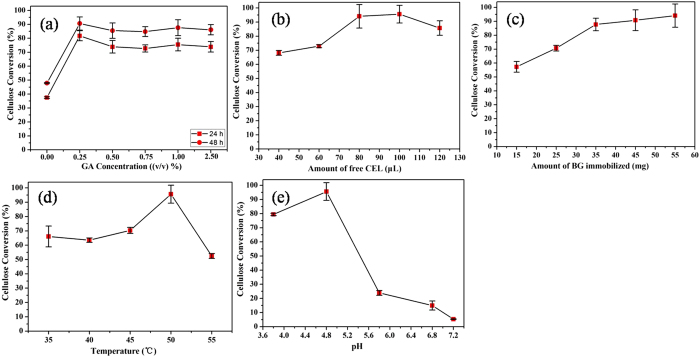
Effect of (**a**) the GA concentration, (**b**) the amount of free CEL, (**c**) the amount of immobilized BG, (**d**) the temperature and (**e**) the pH during the hydrolysis of filter paper in the reaction system (20 mg filter paper and 10 mL citrate buffer (5 mM) for 48 h).

The main function of the BG-loaded fabric was to convert cellobiose into glucose. To obtain the optimum conversion conditions in a situation with filter paper as the cellulose substrate and CEL (ATCC 26921) as the EG and CBH donors, various factors including the effect of the GA concentration, the amount of immobilized BG, the temperature, and the pH were systemically investigated to evaluate the performance of the catalytic fabric. The results are shown in Fig. 6. As we can see from Fig. 6a, GA concentrations ranging from 0% to 2.5% (v/v) were used to cross-link BG, and it was found that 0.25% (v/v) was the optimum GA concentration for cross-linked BG. This indicated that a moderate cross-linking of BG was effective to ensure the retention of the enzyme in the net-cloth. The influence of the amount of CEL and BG on the cellulose conversion are shown in Fig. 6b,c. Taking into account that excess amounts of enzymes may increase the cost of the bioconversion, 100 μL CEL and 55 mg immobilized BG were finally selected. The temperature and the pH dependence of the BG-loaded fabric are shown in Fig. 6d,e. The temperature significantly influenced the hydrolysis reaction by affecting the stability of the BG and the kinetics of the reaction. As shown in Fig. 6d, the optimum temperature was 50 °C. Under optimal temperature conditions, the enzyme-loaded fabric showed a maximum activity at pH 4.8 (Fig. 6e). Beyond that, the BG activity decreased markedly at pH above 5.8. In conclusion, 0.25% (v/v) GA for 55 mg BG treatment, 100 μL free CEL, 50 °C and citric acid buffer (pH 4.8) are the best conditions for the hydrolysis of filter paper with BG-loaded fabric.

**Figure 7:**
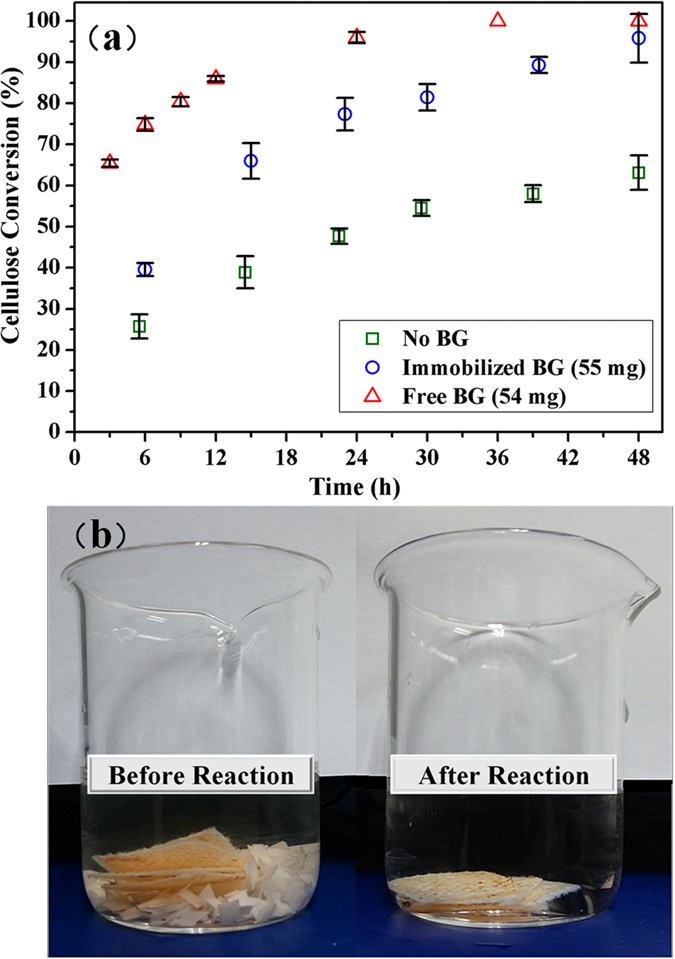
(**a**) The effect of free and immobilized BG on the hydrolysis of filter paper. Reaction mixtures containing 100 μL free CEL and 20 mg filter paper in 10 mL of a 5 mM citric acid buffer (pH 4.8), incubated at 50 °C for up to 48 h. (**b**) Digital camera images of the hydrolysis reaction system at 0 h and 48 h.

The conversion of filter paper into glucose as a function of time by only CEL, CEL plus BG, and CEL plus the immobilized BG is shown in Fig. 7a. From the results, it can be stated that 1) compared with the group without supplementing BG, the conversion of cellulose to glucose increased almost 40% at 48 h after adding the BG-loaded fabric. The hydrolysis of cellulose took place from the synergistic effect of EG, CBH and BG. CEL has all of these three enzymes, and could thus convert 58% cellulose into glucose after 48 h. But the content of BG was much lower than the other two enzymes in the CEL system, which prevented cellobiose from being converted into glucose in the required time^24^. The BG-loaded fabric could further promote the conversion of cellobiose into glucose, thereby significantly improving the conversion. 2) The group with the free BG hydrolyzed the filter paper at a higher rate than the group with the BG-loaded fabric. This was attributed to dynamic factors causing the liquid-liquid reaction to react faster than its liquid-solid counterpart. But the cellulose conversions of both groups finally reached values higher than 90% after hydrolysis for 48 h. Figure 7b represents two digital camera images of the hydrolysis reaction system. After catalysis of free CEL and BG-loaded fabric for 48 h, the bulk, i.e., insoluble filter paper, was finally converted into a clear and transparent glucose solution, while the light yellow BG-loaded fabric remained unchanged. Consequently, this fabric can be easily separated after the reaction for recycling.

**Figure 8:**
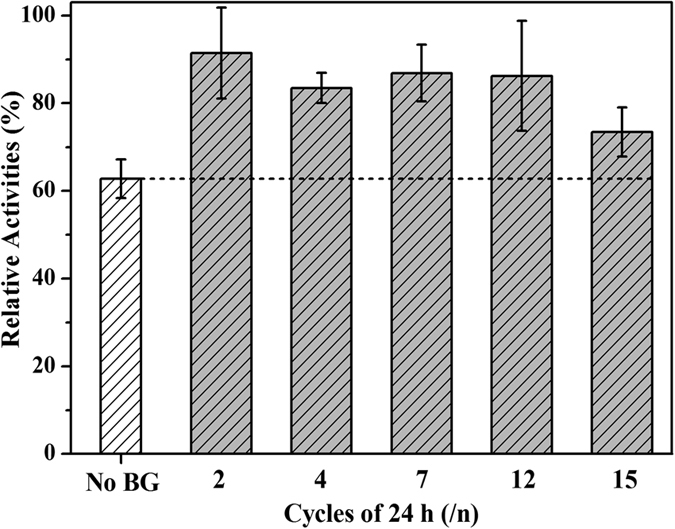
Operational stability of the BG-loaded fabric during hydrolysis of filter paper. The catalytic reaction was performed under optimum conditions (i.e., 100 μL free CEL, 200 rpm, 50 °C and 10 mL citric acid buffer (pH 4.8)). One reaction cycle corresponded to 24 h. BG was cross-linked with 0.25% (v/v) GA.

The ultimate aim of our work is to render the catalytic fabric reusable for a long time and thus make the bioconversion process economically viable. To characterize its operational stability, the reuse of BG-loaded fabric was studied under optimum conditions. As shown in Fig. 8, the operational stability of the BG-loaded fabric was examined during fifteen successive rounds of filter paper hydrolysis, and we found that after fifteen cycles the hydrolysis rate in the presence of BG-loaded fabric still remained higher than in the absence of added BG. This demonstrated a good recovery and operational stability of the immobilized BG over the total 15-day experimental period, which could significantly reduce the production cost of cellulosic ethanol.

In summary, to improve the cellulose conversion yield and make the cellulose hydrolysis process economically viable, a protocol for the *in-situ* encapsulation of BG into a non-swelling PEG net-cloth grafted on a nonwoven fabric by visible light-induced surface controlled/living graft cross-linking polymerization was developed. Because of the favorable reaction conditions of visible light irradiation and room temperature, most of the entrapped BG can retain its activity after the graft cross-linking polymerization. Compared with a catalytic system without a BG-loaded fabric, one with a BG-loaded fabric can give rise to a 40% increase in cellulose conversion. Proper cross-linking of the enzyme limits the leakage of BG from the net-cloth and allows repeated use of the BG-loaded fabric for more than 15 cycles. This strategy has potential applications in bioenergy and biotechnology.

## Methods

### Chemicals and reagents

A PP nonwoven fabric was ordered from Langfang Huijing Paper Plastics Manufacture Inc. This fabric was washed with excess acetone and then dried at room temperature. ITX was obtained from TH-UNIS Insight Co., Ltd. Citric acid monohydrate and trisodium citrate dihydrate was purchased from Sinopharm Chemical Reagent Co., Ltd. Cellulase was obtained from *Trichoderma reesei* (EC 3.2.1.4), *β*-glucosidase from *almonds* (EC 3.2.1.21), PEGDA with a molecular weight of 575 was provided by Sigma-Aldrich Chemical Co. FUN^®^ 1 and Calcofluor^®^ was obtained from Life Technologies Co. Filter paper (used as a cellulose model) was ordered from Hangzhou Special Paper Co., Ltd. Other chemicals were purchased from Alfa Aesar Chemical Co.

### Introducing isopropyl ITXSP groups on the PP nonwoven fabric

ITX can be grafted on a fabric as follows. Firstly, an ITX acetone solution (2 mL, 3 mmol/mL) was uniformly coated onto an area of 4 × 4 cm^2^ of the fabric after which the fabric was placed between two quartz plates. Secondly, the system was irradiated under a high-pressure mercury lamp (wavelength 254 nm, 9 mW/cm^2^) for 6 min at room temperature to obtain a fabric with ITXSP groups. After the irradiation reaction, the fabric with ITXSP was washed with excess acetone to remove the residual ITX. Finally, it was dried at room temperature.

### Preparation of free and cross-linked BG solutions

For the free enzyme solution, 55 mg BG was added to 800 μL of a citric acid buffer (5 mM, pH = 4.8) by shaking. To prepare the cross-linked BG solutions, 0.25% (v/v) of GA was added to the free BG solutions for BG linkage at 30 °C for 30 min.

### *In-situ* BG immobilization on the fabric

Firstly, 800 μL of the BG solution was mixed with 600 μL PEGDA via shaking, after which the solutions were cast onto the fabric-ITXSP. The fabric was sandwiched between two quartz plates to spread the PEGDA/BG solutions evenly. Polymerization was then carried out during 90 min under a xenon lamp (a filter was added with a band-pass of 380–700 nm, the irradiation intensity was 3 mW cm^−2^ at 420 nm). This fabric was then washed with deionized water three times to remove the unimmobilized BG and PEGDA.

To prepare the BG-loaded fabric for the fluorescence investigation, this fabric was immersed in 100 mL Na-HEPES buffer (10 mM, pH = 7.2). Subsequently, 100 μL FUN^®^ 1 and 500 μL Calcofluor^®^ solutions were added to the buffer. After 60 min at room temperature, the BG-loaded fabric was taken out from the solution and thoroughly washed with Na-HEPES buffer to remove the fluorescent agent without interaction. All steps were performed in a darkroom.

### Detection of immobilized BG on the PEG net-cloth

A Non-Interference Protein Assay Kit was used to detect the ratio of immobilized BG in the PEG net-cloth^48^. All of the BG absorbed on the quartz plate and the BG-loaded fabric having undergone the graft reaction were thoroughly rinsed and collected to determine the non-immobilized amount. The BG concentration after immersion in citric acid buffer was measured by monitoring its absorbance at 480 nm using a UV−vis spectrophotometer. The concentration of BG in the solution was obtained from a calibration curve, and the amount of BG released at time t (*M*_t_) was calculated by accumulating the total BG released up to that time. The ratio of released BG, *M*_t_/*M*_0_, could then be calculated. Here, *M*_0_ is the amount of the initially immobilized BG.

### BG activity assays

The activity of the BG-loaded fabric was measured as the amount of *p*-nitrophenol released from *p-*NPG using a calibration curve at 405 nm^49^. The 5 mL reaction mixture containing 4 mM *p-*NPG, 50 mM sodium acetate buffer (pH = 4.8) was first incubated at 40 °C, after which the 2 × 2 cm^2^ BG-loaded fabric was added and the reaction catalyze for 30 min. Finally, the reaction was stopped by adding 10 mL of 1 M Na_2_CO_3_ and the solution was measured at 405 nm. One unit of BG activity was defined as the amount of BG required to catalyze the formation of 1 μmol of *p*-nitrophenol *per* min.

### Hydrolysis of cellulose

The hydrolytic activity of the BG-loaded fabric was tested in several cycles (24 h each) at 50 °C and pH 4.8. Firstly, 100 μL free CEL and the 4 × 4 cm^2^ BG-loaded fabric were mixed in 10 mL citrate buffer (5, pH 4.8) after which 20 mg of filter paper was added to the buffer. Secondly, the mixture was stirred at 200 rpm and 50 °C for 24 h. Finally, the BG-loaded fabric was taken out and thoroughly washed with citrate buffer at 50 °C in order to be reused in the next cycle. The liquid phase was sampled after each hydrolytic cycle to determine the concentration of the glucose yield.

### Instruments and characterization

The UV spectra were characterized by a UV−vis spectrophotometer (U-3900H from HITACHI Co.). The contact angle data was obtained with an OCA 20 tensiometer from Dataphysics Co. SEM (JSM-6701F from JEOL Japan Electronics Co., Ltd) was used to determine the morphology of the non-woven fabric before and after grafting reactions. The structure of this catalytic composite was characterized by microscope (BX 53 from Olympus Co.) and atomic force microscope (AFM, CP-II from VEECO Co.). The surface chemistry was probed by XPS (ESCALAB 250 from Thermo Fisher Scientific Co.) with a monochromator. The BG-loaded fabric with fluorescence was characterized with a CLSM (TCS SP8 from LEICA Co.). The concentration of glucose was determined with a biosensor (SBA-40D from Biology Institute of Shandong Academy of Sciences).

## Additional Information

**How to cite this article**: Zhu, X. *et al*. Net-Immobilization of *β*-glucosidase on Nonwoven Fabrics to Lower the Cost of “Cellulosic Ethanol” and Increase Cellulose Conversions. *Sci. Rep.* **6**, 23437; doi: 10.1038/srep23437 (2016).

## Supplementary Material

Supplementary Information
