# Supplementary material for: Net-Immobilization of β-glucosidase on Nonwoven Fabrics to Lower the Cost of “Cellulosic Ethanol” and Increase Cellulose Conversions
Source: Sci Rep. 2016 Mar 24;6:23437. doi: 10.1038/srep23437 (PMC4806303; doi:10.1038/srep23437)
Supplement: Supplementary Information [file srep23437-s1.doc]

**Supporting Information**

Net-Immobilization of β-glucosidase on Nonwoven Fabrics to Lower the Cost of “Cellulosic Ethanol” and Increase Cellulose Conversions

Xing Zhu,^1,2^ Bin He,^1,2^ Changwen Zhao,*^,1,2^ Rong Fan,^1,2^ Lihua Zhang,^1,2^ Guan Wang,^1,2^ Yuhong Ma,^3^ Wantai Yang*^, 1,2,3^

^1^ State Key Laboratory of Chemical Resource Engineering, Beijing University of Chemical Technology, Beijing 100029, China

^2^ Beijing Laboratory of Biomedical Materials, Beijing University of Chemical Technology, Beijing 100029, China

^3^ Key Laboratory of Carbon Fiber and Functional Polymers, Ministry of Education, Beijing University of Chemical Technology, Beijing 100029, China

Correspondence and requests for materials should be addressed to

W.T.Y. ([yangwt@mail.buct.edu.cn](mailto:yangwt@mail.buct.edu.cn))

C.W.Z. ([zhaocw@mail.buct.edu.cn](mailto:zhaocw@mail.buct.edu.cn))

To further analyze the BG loaded fabric, the structure of this composite was characterized by the digital camera, microscope and atomic force microscope (AFM), respectively. The blank non-woven fabric (Fig. S1a), the fabric-*g*-P(PEGDA) (Fig. S1b) and the BG loaded fabric (Fig. S1c) were stained by Coomassie brilliant blue and then washed with excess ethanol and acetone. Coomassie Brilliant Blue is one dye that is commonly used for staining proteins in analytical biochemistry. These fabrics were then further detected by the microscope. Compared with the morphology of the blank non-woven fabric (Fig. S1d), the PEG net-cloth grafted on and within the fabric can be observed obviously in Fig. S1e and Fig. S1f. Due to that Coomassie brilliant blue can’t specifically interact with PEG networks, the fabric-*g*-P(PEGDA) (the fabric grafted PEG net-cloth without loading BG) can not be dyed blue (Fig. S1b and Fig. S1e). While the blue regions in Figure S1c and Fig. S1f indicated that the enzymes were embedded into the PEG net-cloth successfully. What’s more, typical AFM images for these three fabrics were also investigated. Fig. S1g demonstrated that the diameter of the fabric’s fiber was about 10 μm. Although XPS characterization proved that there were some enzymes studded on the surface, no obvious difference was detected between Fig. S1h and Fig. S1i which indicated that most of the BGs were entrapped into the smooth and dense net-cloth.


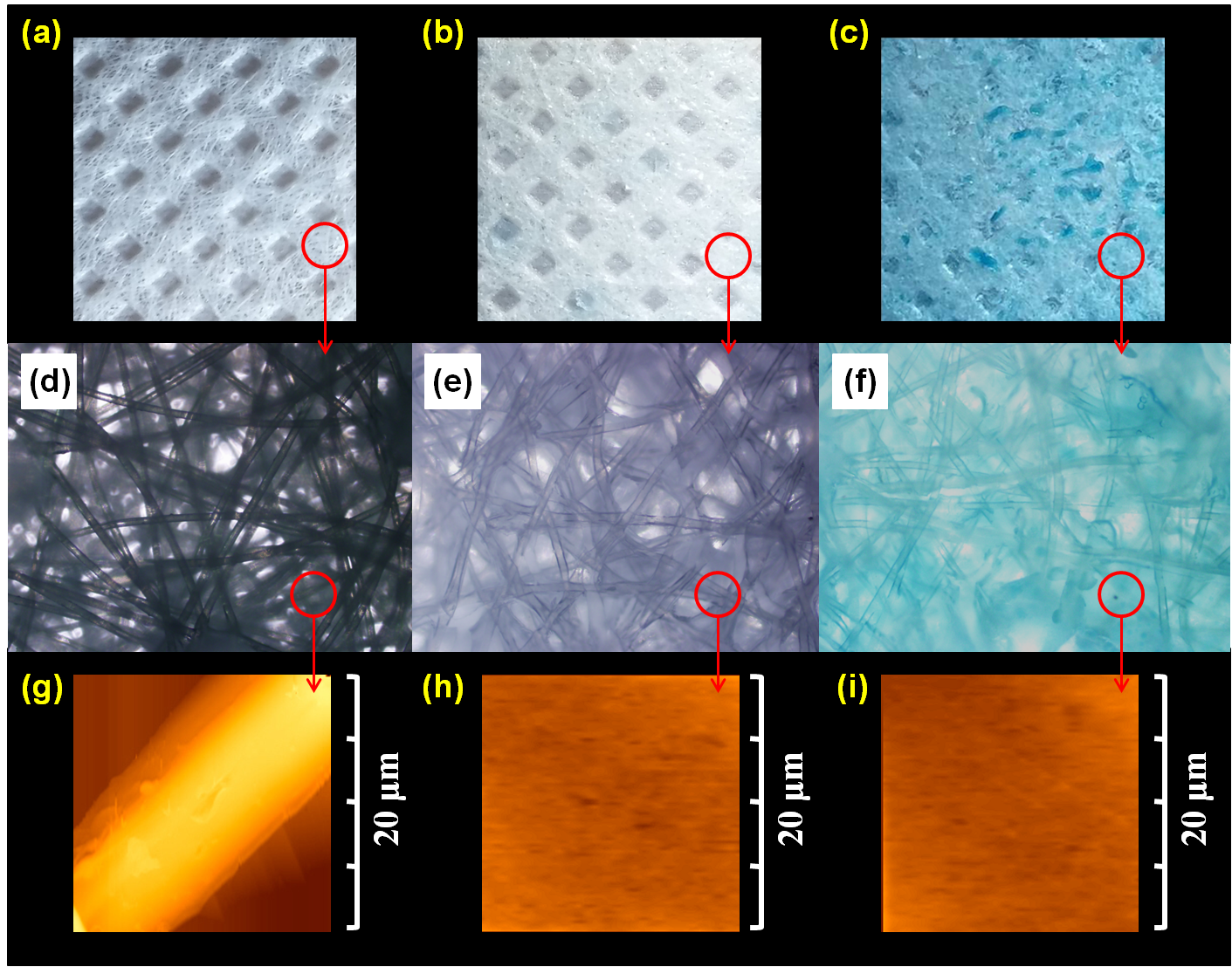


**Figure S1. Digital photo of** (**a**) the blank non-woven fabric, (**b**) the fabric-*g*-P(PEGDA) and (**c**) the BG loaded fabric. Microscope images of (**d**) the blank non-woven fabric, (**e**) the fabric-*g*-P(PEGDA) and (**f**) the BG loaded fabric. Typical AFM images of (**g**) the blank non-woven fabric, (**h**) the fabric-*g*-P(PEGDA) and (**i**) the BG loaded fabric. All the fabrics were stained by Coomassie brilliant blue for 1 h and then washed with excess ethanol and acetone.
